# Targeting Aurora A Kinase Enhance the CDK4/6 Inhibitor Sensitivity in HR+/HER2- Breast Cancer

**DOI:** 10.32604/or.2026.081653

**Published:** 2026-07-16

**Authors:** Juan Wu, Yue Wang, Honglin Yan, Juanjuan Li, Chuntao Quan, Jingping Yuan, Shengrong Sun

**Affiliations:** 1 Department of Pathology, Renmin Hospital of Wuhan University, Wuhan, China; 2 Department of Breast and Thyroid Surgery, Renmin Hospital of Wuhan University, Wuhan, China; 3 Department of Oncology Medicine, Central People’s Hospital of Zhanjiang, Zhanjiang, China; 4 Department of Pathology, Biobank, Shenzhen Second People’s Hospital, Shenzhen University, Shenzhen, China

**Keywords:** CDK4/6i resistance, spindle assembly checkpoint, Aurora A kinase, RB phosphorylation, ubiquitination

## Abstract

Objectives: Despite the success of CDK4/6 inhibitors (CDK4/6i) in treating HR+/HER2- breast cancer (BC), some patients experience treatment failure due to CDK4/6i resistance. This study aimed to investigate whether targeting Aurora A kinase enhances CDK4/6 inhibitor sensitivity. Methods: An Abemaciclib-resistant cell line (MCF7AR) was developed by treating MCF7 cells with gradually increasing concentrations of Abemaciclib. We evaluated the relative protein levels of p-RB, p-Aurora A, Aurora A, and USP22 in cell cultures, animal tissues, and clinical samples. The effect of Aurora A inhibition on reversing CDK4/6i resistance was assessed using cell viability assays and tumor xenograft experiments. We examined the relationship between Aurora A kinase activation levels and resistance to CDK4/6i. Results: CDK4/6i-resistant cell lines and patient samples exhibited elevated levels of phosphorylated Aurora A and retinoblastoma protein (RB). Previous studies have reported that RB inactivation can activate the spindle assembly checkpoint (SAC), leading to mitotic delay. High Aurora A activity counteracts the SAC-induced delay, thereby promoting mitosis. CDK4/6i treatment increased Aurora A protein levels through regulation by USP22, enhancing Aurora A activity and overcoming SAC-mediated cell cycle arrest. Combined therapy with Aurora A inhibitor (Aurora Ai) and CDK4/6i demonstrated synergistic antitumor effects both *in vitro* and *in vivo*. Clinical data suggest that HR+/HER2- patients with high levels of phosphorylated RB and Aurora A may exhibit resistance to CDK4/6i. Conclusion: Aurora A contributes to CDK4/6i resistance by overcoming SAC delay and promoting mitosis. In RB-inactivated CDK4/6i-resistant cells, Aurora A inhibition may induce a synthetic lethal effect.

## 1 Introduction

Breast cancer is the most common type of cancer among women, significantly affecting their well-being [1]. The HR+/HER2- subtype constitutes approximately 60%–70% of all breast cancer cases [2]. Recently, targeting the CDK4/6 protein has emerged as a key focus in breast cancer research. CDK4/6i (abemaciclib, palbociclib, and ribociclib) effectively inhibit the kinase activity of CDK4 and CDK6 in breast cancer cells, halting the phosphorylation of the Rb protein and thereby preventing the G1/S phase transition of the cell cycle. Despite the notable success of CDK4/6 inhibitors in treating advanced HR+/HER2- breast cancer [3,4], some patients encounter treatment failure due to either primary or acquired resistance to these inhibitors [5,6]. Resistance to CDK4/6i significantly contributes to the morbidity and mortality of patients with HR+/HER2- breast cancer. Initially, many patients develop primary resistance to CDK4/6i. Subsequently, those who initially respond to treatment inevitably develop acquired drug resistance and experience disease progression over time [7]. Moreover, nearly all patients receiving CDK4/6i treatment develop drug resistance as the treatment duration extends [8]. Therefore, in-depth exploration of the mechanisms underlying CDK4/6i resistance is imperative for clinicians to assess novel treatment approaches.

The resistance mechanisms of CDK4/6i mainly include continuous expression of bypass signaling molecules and cell cycle proteins, primarily E2F, CDK4, CDK6, and Cyclin E gene amplification [9,10,11,12]. Following phosphorylation by CDK4/6 kinases, the RB protein becomes inactive, leading to the dissociation of RB and E2F, which controls downstream cyclin transcription and propels the cell cycle forward. Studies have indicated that CDK4/6i resistance mechanisms encompass bypassing reliance on the CDK4/6 pathway, characterized by cyclin E1 amplification and RB deletion [6]. RB1 deletion further facilitates E2F dissociation and G1/S cell cycle transition [13]. Loss of RB expression has been identified in cellular models resistant to CDK4/6i, with rare RB1 alterations detected in tumor samples and circulating tumor DNA (ctDNA) from patients with CDK4/6i exposure [14,15]. RB phosphorylation typically results in its inactivation, driving the G1/S transition. Activation of the MAPK and PI3K-AKT signaling pathways mediates CDK4/6i resistance, primarily through key molecules PTEN and PDK1 in downstream pathways [16,17,18]. Reports suggest that AKT in combination with CDK4/6i can effectively overcome CDK4/6i resistance [19]. Clinical samples from CDK4/6i-resistant patients have shown overexpression of cyclins E1 and E2, indicating their potential role in drug resistance. Further experimental validation is required to confirm the significance of cyclins E1 and E2 in driving resistance [20,21,22]. Preclinical and clinical data have linked alterations in ERBB2 and FGFR2 to CDK4/6i resistance [23,24]. Targeting the ACAA1 protein has been shown to enhance sensitivity to CDK4/6 inhibitors [25,26]. A novel Antibody-Drug Conjugate (ADC) drug antibody, gossamer (SG), targeting the cell surface antigen Trop-2, has been developed and administered to patients with advanced breast cancer who experienced disease progression after CDK4/6i treatment [27]. Targeting the autophagy pathway may improve clinical outcomes in patients with CDK4/6i treatment failure in advanced breast cancer [28]. Additionally, targeting CDK7 enhances the antitumor effects of CDK4/6i [29].

Aurora kinases are a class of highly evolved and conserved serine/threonine kinases with subtypes A, B, and Aurora-C subtypes. They are dynamically distributed throughout the cell cycle and regulate processes such as the maturation and separation of the centrosome during cell division, and the assembly and stability of bipolar spindles [30]. Aurora A, initially localized in centrioles at the S phase onset, triggers mitosis and peaks in expression and activity during the G2/M transition [30,31]. Precise chromosome separation and cytoplasmic division are essential for completing the cell cycle successfully. Recent studies have revealed that Aurora kinases are often overexpressed in solid human tumors, particularly Aurora A, leading to abnormal cell division, genetic instability, and influencing tumor occurrence, proliferation, and prognosis [32,33]. Aurora A modulates survivin expression via the FOXP1–FBXL7 ubiquitination complex, contributing to drug resistance in gastric cancer cells and promoting their proliferation [34], presenting a novel antitumor target. In this study, we report that the RB protein is consistently phosphorylated and inactivated in CDK4/6i-resistant cells. What is the relationship between RB inactivation and cell-cycle progression? RB deficiency or inactivation is a common phenomenon [35], and RB deficiency leads to the continuous activation of SAC-associated proteins in tumor cells [36]. The phosphorylation levels of the SAC-related proteins TKK and CASC5 increased upon SAC activation, and the mitotic checkpoint complex (MCC) inhibited the activity of the E3 ligase CDC20, thereby inhibiting the degradation of Cyclin B and blocking the mitotic process [36]. RB inactivation by phosphorylation promotes mitotic arrest, and what force enables drug-resistant cell strains to overcome mitotic delay caused by SAC activation? The activity of Aurora A gradually increased during the development of drug-resistant cell lines. Highly active Aurora A accelerated spindle formation and promoted mitosis, overcoming the mitotic delay effect caused by SAC activation, which leads to greater “addiction” of CDK4/6i cell proliferation to the activity of Aurora A.

Investigating the molecular mechanisms underlying Aurora A’s role in reversing CDK4/6i resistance can offer insights for devising innovative clinical treatment strategies. We aimed to investigate the role of Aurora A kinase inhibition in overcoming resistance to CDK4/6 inhibitors and to explore the potential clinical application of Aurora A inhibitors. Additionally, we explored the relationship between RB phosphorylation and Aurora A activity in CDK4/6i-resistant cells and clinical samples. We discovered that inhibiting Aurora A can produce a synthetic lethal effect, slowing the mitotic process and killing tumor cells. Notably, the Aurora A inhibitor Alisertib, developed by Takeda, is currently undergoing phase II/III clinical trials. This study may improve the application of the Aurora A inhibitor Alisertib in overcoming resistance to CDK4/6 inhibitors.

## 2 Materials and Methods

### 2.1 Cell Culture and Reagents

The MCF7(CL-0149), MDA-MB-231(CL-0150), HCC1937(CL-0093), T47D(CL-0228), 293T(CL-0005), and HCC1806(CL-0729) cells were obtained from Procell Biotechnology Company (Wuhan, Hubei, China). All cell lines were free of mycoplasma contamination and underwent STR authentication. The MCF7AR was obtained by continuously increasing the concentration of Abemaciclib (Ambeed, A106236, Chicago, IL, USA) on the parental MCF7 cells. All cells were cultured in Dulbecco’s Modified Eagle’s Medium (DMEM) (Hyclone, SH30243.01, Shanghai, China) supplemented with 10% fetal bovine serum (FBS) (Excell, FSP500, Suzhou, China) and 1% antibiotic (Gibco, 15240062, Shanghai, China) at 37°C with 5% CO_2_. Cells were used for no longer than 12 months before being replaced. The inhibitors Abemaciclib, Alisertib(A102516), and Usp22i-S02(A1938695) were purchased from Ambeed, Inc. (Chicago, IL, USA). Cycloheximide (CHX, HY-12320) was purchased from MedChemExpress, Inc. (Monmouth Junction, NJ, USA). The antibody information for inhibitors was listed in Supplementary Table S1. The antibodies p-RB(#8516), RB(#9309), p-CASC5(#40758), p-H3(#9701), p-Aurora A(#3079), Aurora A(#14475), USP22(#52293), CDK4(#12790), and CDK6(#13331) were obtained from Cell Signaling Technology, Inc. (Danvers, MA, USA). The antibodies GAPDH(10494-1-AP), Flag(66008-4-AP), H3(17168-1-AP), and SMC3(14185-1-AP) were purchased from Proteintech, Inc. (Wuhan, Hubei, China). The antibody p-SMC3(AWA57326) was purchased from Abiowell, Inc. (Changsha, Hunan, China). The antibody p-TTK(#AF8194) was purchased from Affinity, Inc. (Cincinnati, OH, USA). The antibody information for WB and IHC was listed in Supplementary Tables S1 and S2.

### 2.2 Generation of Abemaciclib Resistance Cell Lines

MCF7 cells were gradually exposed to increasing concentrations of Abemaciclib, starting at 0.1 μM and ultimately reaching 20 μM, leading to the development of the MCF7 Abemaciclib-resistant (MCF7AR) strain. On day 1, after treating MCF7 cells with Abemaciclib for 3 days, the cells were transferred to a drug-free DMEM medium (HyClone, SH30243.01) for 4 days to allow the population to recover to 80% confluence. On day 7, the drug concentration was increased to 0.3 μM for 3 days, followed by 7 days in drug-free medium to restore cell density. On day 20, the Abemaciclib concentration was raised to 1 μM and maintained for 7 days, after which the cells were allowed to recover to 80% confluence. By day 30, the cells proliferated stably at 1 μM. On day 60, the concentration was increased to 5 μM, the drug was removed after 3 days of treatment, and the cells resumed normal growth at 5 μM. On day 90, the cells grew normally at 10 μM for approximately 30 days. Finally, on day 120, the cells continued to proliferate at 20 μM for 60 days, resulting in the stable proliferation of MCF7 AR cells at this concentration.

### 2.3 CCK-8 Assays and Cell Crystal Violet Staining Assay

The CCK8 assay was performed according to the manufacturer’s protocol (Beyotime, C0038, Shanghai, China). MCF7AR cells were initially seeded in 96-well plates at a density of approximately 1 × 10^4^ cells per well, treated with Abemaciclib and Alisertib at varying concentrations ranging from 0.01 to 10 μM, and incubated at 37°C. The volume of CCK-8 reagent is usually 1/10 of the total. After incubating the CCK-8 reagent for 2 h, cell viability was measured compared with the blank control wells by absorbance at 450 nm using a microplate reader (Thermo Fisher Scientific, Multiskan Ease, Shanghai, China). The cell crystal violet staining was used for the assessment of cell viability. MCF7AR cells were plated on a 12-well dish starting at a density of approximately 5 × 10^4^ cells per well, and 3 parallel wells were used to improve the credibility of the experiment. The tumor clones formed after drug treatment in about 10–14 days. After applying 4% PFA for 10 min of fixation, the cells were stained with 0.2% crystal violet solution (Beyotome, C0121, Shanghai, China) for 15 min. The pictures of tumor clones were captured after removing the remaining residue.

### 2.4 Western Blot (WB) and Immunohistochemistry (IHC)

Cells were harvested using RIPA (Beyotome, P0013B, Shanghai, China) buffer (including protease and phosphatase inhibitors) for 30 min on ice, and protein concentration was determined by BCA assay (Beyotome, P0012, Shanghai, China). 5× loading buffer (Beyotime, P0015L, Shanghai, China) was used for protein denaturation, and equal amounts of 30 μg protein were separated by 10–12% SDS-PAGE and transferred to PVDF membranes. The membranes were blocked in 5% skim milk for 2 h at room temperature and incubated with primary antibodies at 4°C overnight. The membranes were incubated with 1:1000 diluted secondary antibodies (Beyotime, A0354/A0208, Shanghai, China) for about 2 h after washing three times for 5 min each with TBST at room temperature. The image can be obtained by the chemiluminescent detection system (Tanon, 5200Mui, Shanghai, China).

5 μm slides were first dewaxed and hydrated in the IHC assay. The antigen retrieval process was completed by adding a 10 mM citrate buffer solution and heating it with microwaves until boiling for 10 to 20 min. Then, endogenous peroxidase was inactivated by incubating the sample in a 3% H_2_O_2_ aqueous solution at room temperature for 5 min. Finally, a 5% BSA solution was used to block non-specific binding sites for 1 h at room temperature. Subsequently, sections were incubated with primary antibodies overnight at 4°C, followed by incubation with 1:100 diluted secondary antibodies (Beyotime, A0354/A0208, Shanghai, China) for 30 min at room temperature. The images for IHC can be obtained after DAB staining (ZSGB-BIO, ZLI-9017, Beijing, China). The DAB staining process takes approximately 0.5 to 2 min, and the positive signal, indicated by brownish particles, can be observed under a microscope (Nikon, Eclipse TS-100F, Tokyo, Japan).

### 2.5 Human Tissue Specimens

Paraffin-embedded specimens from 98 BC patients were collected at Renmin Hospital of Wuhan University. These BC patients had received CDK4/6i treatment within the past five years. The collection of BC samples was conducted in accordance with established guidelines. All BC sample acquisitions were approved by the Committee on Ethics of Renmin Hospital of Wuhan University (WDRY2025-K331). Written informed consent was obtained from all participants prior to the experiment, and the study was conducted in accordance with the Declaration of Helsinki. The pathological information of BC patients was listed in Supplementary Table S3.

### 2.6 Plasmid and shRNA/siRNA Primers

The construction of WT/S807A-RB, shRB, shAURKA, WT-AURKA, WT-USP22, and shUSP22 plasmids was completed by Dingke Biotechnology Company in Shenzhen. These plasmids were transfected into MCF7/MCF7AR cells using Lipofectamine 3000 reagent (Thermo Fisher Scientific, L300001, Shanghai, China), according to the ratio of Lipo3000 to plasmid is 30 μL:10 μg. The ratio of the lentiviral packaging plasmids for the stable knockdown cell line is shRNA:psPAX2:pMD2.G = 4:3:1. For example, the cell density reached 80% in a 6 cm dish before transfection, and the amount of DNA used for transfection was approximately 10 μg. Primer information was listed in Supplementary Table S4.

### 2.7 Immunofluorescence (IF) and Multiple Immunofluorescent Assay (mIF)

Cells were fixed with 4% paraformaldehyde for 5 min. After permeabilization with 0.2% Triton X-100 (Solarbio, IR9071, Beijing, China), cells were blocked with 5% BSA for 2 h at room temperature, then incubated with the primary antibody at 4°C overnight, followed by incubation with a fluorescent secondary antibody (Bioss, bs-0295G, Beijing, China) diluted 1:200 at room temperature for 2 h. Fluorescent images were captured by a fluorescence microscope (Nikon, Eclipse TS-100F, Tokyo, Japan). Slides were stained with each antibody and fluorescent dyes in sequence for the mIF assay, then incubated with corresponding fluorophore-conjugated secondary antibodies for 10 min. Lastly, the tyramide signal amplification reaction was completed by adding the corresponding substrates (Huilan Biotech, RC0086plus-45RM, Shanghai, China), at a working dilution of 1:200. The reaction was then incubated at room temperature for 10 min. The antibody information for the immunofluorescence assay was listed in Supplementary Table S2.

### 2.8 EdU-Cell Proliferation Detection

EdU, a thymidine analogue, incorporates into replicated DNA by replacing thymine during cell proliferation. MCF7AR cells were seeded in 96-well plates at a density of 5 × 10^3^ cells per well and cultured overnight; each well contained 100 μL of medium. Then, 100 μL of EdU (Beyotime, ST067, Shanghai, China) was added and incubated for 2 h at 37°C. The final working concentration of EdU in the medium was 10 μM. Then, the cells were fixed with 4% paraformaldehyde at room temperature for 30 min, and permeabilized with 0.5% Triton X-100 at room temperature for 15 min. Afterward, the images of EdU-positive cells were captured using a fluorescence microscope (Olympus Corporation, CKX53, Tokyo, Japan).

### 2.9 Flow Cytometry for Cell Cycle and Apoptosis

The ratios of cells in the G1, S, and G2 phases were analyzed by flow cytometry. Cells were harvested and resuspended in ice-cold 70% ethanol. After staining with a final concentration of 50 μg/mL propidium iodide (PI) (Uelandy, C6078, Suzhou, China) and 100 μg/mL RNase inhibitor, the cells were incubated at 37°C for 30 min in the dark. The percentage of cells in each phase was calculated and presented as the cell cycle profile. The harvested cells were washed twice with cold PBS and resuspended in binding buffer. The cells were then stained with Annexin V-FITC and PI for the apoptosis assay (Uelandy, F6012L, Suzhou, China) and incubated at room temperature in the dark for 15–20 min. The percentage of apoptotic cells was calculated for each group. All stained cells were analyzed using a BD Flow Cytometer (BD, FACS Aria Fusion, New Jersey, USA). Data were analyzed by FlowJo software version 10.10 (BD, New Jersey, USA).

### 2.10 Nude Mouse Xenograft

The purpose of this animal experiment is to investigate whether the combined use of Abemaciclib and Alisertib can exert a synergistic anti-tumor effect. Thirty 4-week-old BALB/c nude mice, weighing 16–18 g, were purchased from Wuhan Shulaibao Biotechnology (China). MCF7AR cells were subcutaneously injected into the nude mice, with a cell count of no less than 2 × 10^6^ per inoculation. Tumor volumes (mm^3^) were calculated using the formula: V = 1/2 × a × b^2^ (a = length, b = width). When the tumor volume reaches 100 mm^3^, all the nude mice were randomly divided into four groups (at least six mice/group). The Abemaciclib and Alisertib were dissolved in a carboxymethylcellulose sodium (CMC-Na) solution (Selleck, S6703, Shanghai, China). Abemaciclib was administered intragastrically at a dose of 50 mg/kg per mouse, and Alisertib was administered intragastrically at a dose of 20 mg/kg per mouse. Drug treatments were given three times per week. Weight-based stratified randomization was employed, using random number tables within each stratum to assign animals randomly to the experimental and control groups. All animal handling, data collection, and result evaluation were conducted in a blinded manner with respect to group assignments. All *in vivo* experiments were approved by the Institutional Animal Care and Use Committee of Shenzhen University (SZU-IACUC-2026-0027) and followed the Guide for the Care and Use of Laboratory Animals.

### 2.11 Statistical Analysis

Results were expressed as the mean ± SEM and were analyzed using a two-tailed Student’s *t*-test, two-way analyses of variance, and ANOVA analyses. Pearson correlation coefficient analysis was performed using GraphPad Prism V5.0 (GraphPad Software, Inc., La Jolla, CA, USA). Differences were considered statistically significant when *p* < 0.05.

## 3 Results

### 3.1 Continuous Phosphorylation of RB Is Associated with CDK4/6i Resistance

**Figure 1:**
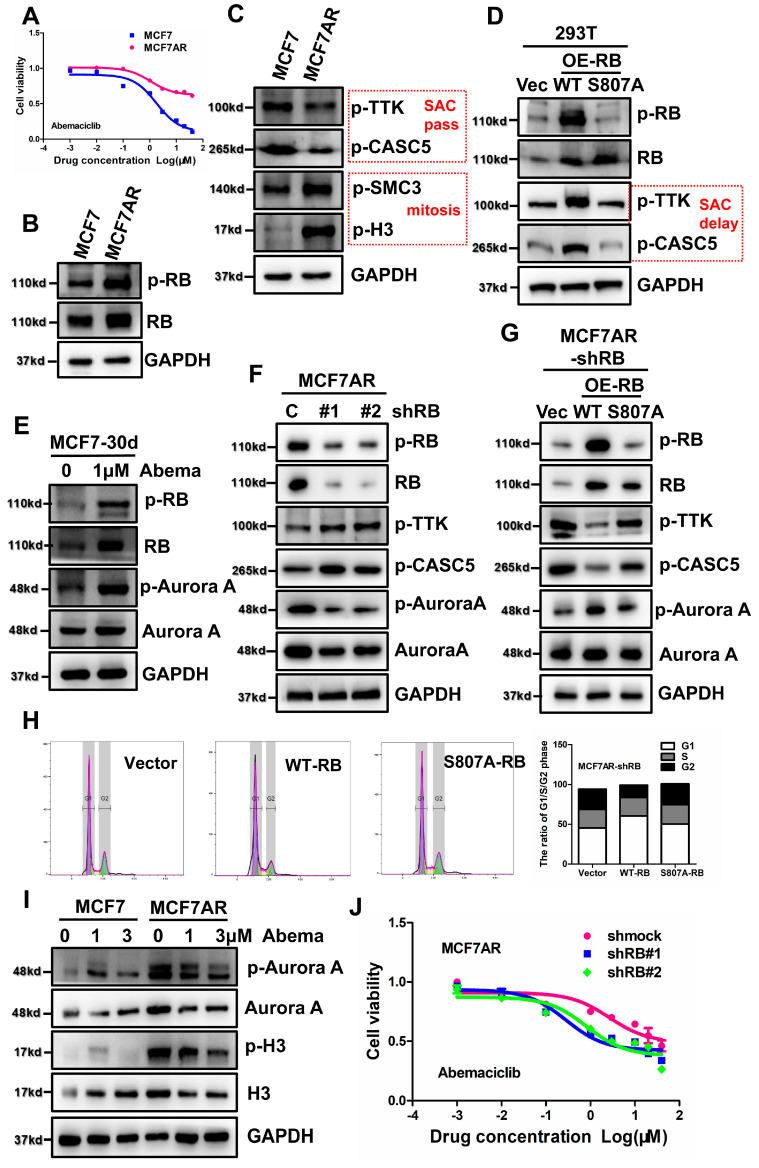
**Continuous phosphorylation of RB is associated with CDK4/6i resistance.** (**A**) The CCK8 assay was used to confirm the successful establishment of the MCF7AR cell lines. (**B**) Protein levels of p-RB and RB were measured in MCF7AR cells compared to parental MCF7 cells. (**C**) Protein levels of p-TTK, p-CASC5, p-SMC3, and p-H3 were measured in MCF7AR cells compared to parental MCF7 cells. (**D**) The levels of p-RB, p-TTK, and p-CASC5 were assessed following overexpression of the WT-RB or S807A-RB mutant plasmid in 293T cells. (**E**) In MCF7 cells treated with Abemaciclib for 30 days (MCF7-30d), the levels of p-RB/RB and p-Aurora A/Aurora A were evaluated. (**F**) Protein levels of p-RB, RB, p-TTK, p-CASC5, and p-Aurora A were also measured in shRB-MCF7AR cells. (**G**) Protein levels of p-RB, RB, p-TTK, p-CASC5, p-Aurora A, and Aurora A were analyzed after overexpression of WT-RB or S807A-RB plasmids in shRB-MCF7AR cells. (**H**) Cell cycle distribution (G1, S, and G2 phases) was determined by flow cytometry following overexpression of WT-RB or S807A-RB plasmids in shRB-MCF7AR cells. (**I**) The levels of p-Aurora A/aurora A and p-H3/H3 were compared between MCF7AR and MCF7 cells under CDK4/6i treatment. (**J**) CDK4/6i sensitivity was assessed using the CCK8 assay in shRB-MCF7AR cells. The results presented have been repeated in 3 biological replicates. Data means ± SEM.

The mechanism of CDK4/6i resistance in patients with HR+/HER2- breast cancer is not yet fully understood. To further investigate the underlying CDK4/6i resistance mechanism, the CDK4/6i Abemaciclib-resistant cell lines (AR, Abemaciclib-resistant) were utilized in this study. The MCF7AR cell line was confirmed to be resistant to Abemaciclib, with an IC50 of 10 μM, in contrast to the IC50 of 1 μM in the MCF7 parental cell line (Fig. 1A). Western blot analysis revealed a upregulation of p-RB in MCF7AR cells compared to MCF7 (Fig. 1B). Additionally, the levels of p-TTK and p-CASC5, biomarkers of SAC activation during RB deficiency [25], were decreased (Fig. 1C). RB phosphorylation resulted in a similar mitotic delay effect as RB deficiency; the levels of p-TTK and p-CASC5 increased when the overexpression of WT-RB in 293T cells compared to S807A-RB overexpression (Fig. 1D). SAC blockage due to RB phosphorylation was not observed in the MCF7AR cell line. What forces drive cells to overcome SAC-mediated cell cycle arrest? The levels of p-SMC3 and p-H3 play a role in promoting mitosis by inducing chromatin condensation and the separation of sister chromatids, respectively, and are increased in MCF7AR cells. Furthermore, we found that the phosphorylation of RB gradually increased during Aurora A activation in MCF7 cells treated with CDK4/6i for 30 days, revealing that the phosphorylation of RB and Aurora A occurs during the development of CDK4/6i resistance (Fig. 1E). Increased p-TTK and p-CASC5 levels were observed in RB-knockdown MCF7AR cells, and reduced p-Aurora A expression was observed (Fig. 1F). In addition, the results showed that overexpression of WT-RB caused a decrease in p-TTK/p-CASC5 and an increase in p-Aurora A in RB-knockdown MCF7AR cells (Fig. 1G). The cell cycle assay showed that the proportion of cells in G2 phase decreased when WT-RB was overexpressed, indicating that the phosphorylation status of RB correlated with G2 phase arrest (Fig. 1H). Considering the role of Aurora A kinase in promoting the transition from the G2 to M phase, we speculated that Aurora A might be the key factor driving CDK4/6i-resistant cells to bypass the SAC checkpoint delay effect. The level of p-Aurora A was slightly affected in MCF7AR cells when CDK4/6i treatment was compared to that in MCF7 cells (Fig. 1I). CDK4/6i sensitivity increased slightly in shRB-MCF7AR cells (Fig. 1J).

### 3.2 High Aurora Activity Is Linked to CDK4/6i Resistance in RB-Phosphorylated Cells

**Figure 2:**
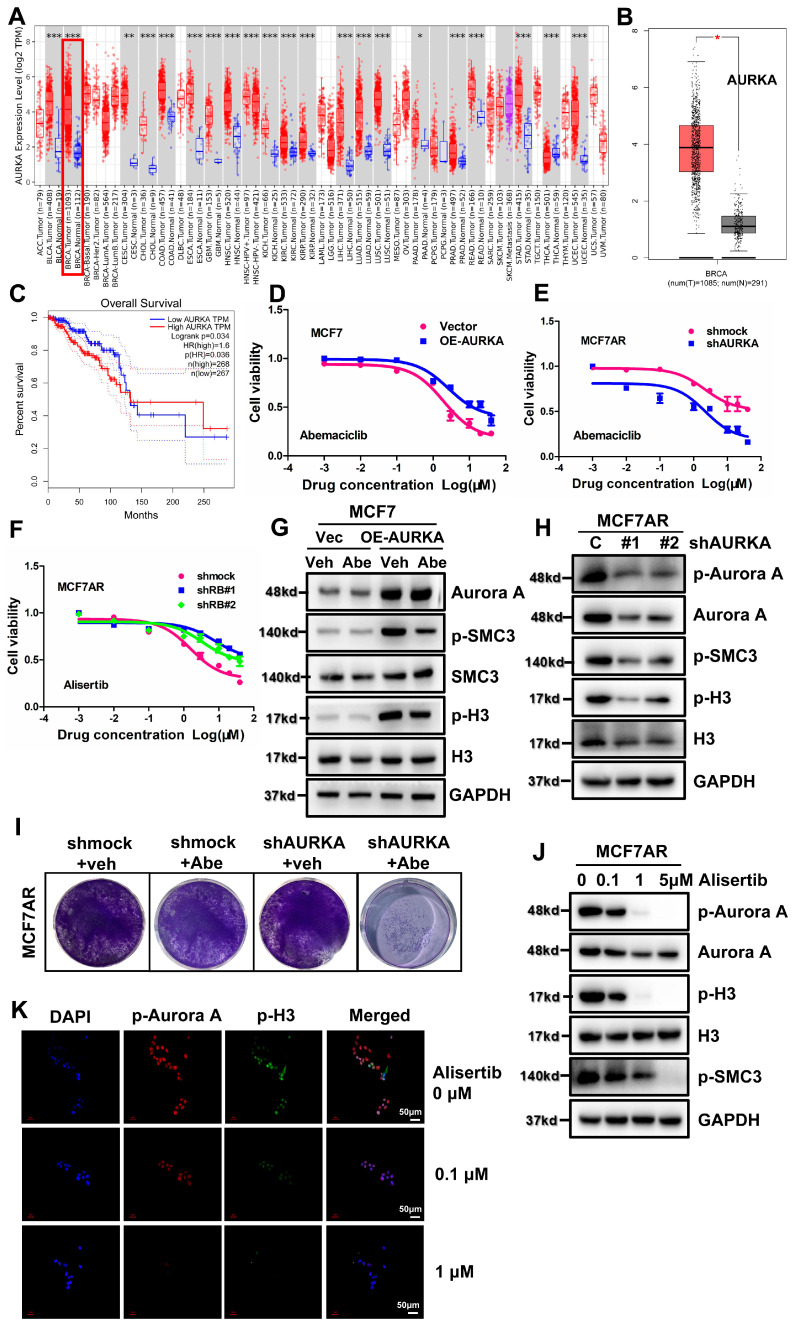
**High Aurora activity is linked to CDK4/6i resistance in RB-phosphorylated cells.** (**A**) Aurora A is highly expressed in most tumor types, according to the GEPIA database. The red scattered dots within the red rectangular frame represent breast cancer tissue, while the blue dots represent normal tissue. (**B**) Aurora A is highly expressed in breast cancer. (**C**) High expression of Aurora A is closely correlated with patient survival rates. (**D**) CDK4/6i sensitivity was assessed under conditions of Aurora A overexpression in MCF7 cells. (**E**) CDK4/6i resistance was checked when Aurora A was knocked down in MCF7AR cells. (**F**) MCF7AR cells exhibited sensitivity to the Aurora A inhibitor Alisertib, with an IC50 of 2 μM; sensitivity was further evaluated using a CCK8 assay in RB-knockdown MCF7AR cells. (**G**) Protein levels of p-SMC3 and p-H3 were analyzed following Aurora A overexpression. (**H**) Protein levels of p-SMC3 and p-H3 were examined when Aurora A was knocked down. (**I**) CDK4/6i sensitivity was examined by the Crystal Violet assay after Aurora A knockdown. (**J**) Protein levels of p-SMC3 and p-H3 were checked after Aurora Ai treatment. (**K**) Fluorescence intensities of p-Aurora A and p-H3 were measured following Aurora Ai treatment. The results presented have been repeated in 3 biological replicates. Data means ± SEM. *p < 0.05, **p < 0.01, ***p < 0.001.

Using the GEPIA database, we discovered that Aurora A is highly expressed in various tumor types; the red rectangular box indicates that the expression level of AURKA is higher in breast cancer tissues than in normal breast tissues (Fig. 2A,B). Its high expression is closely linked to survival rates (Fig. 2C). Aurora A overexpression increased CDK4/6i resistance in MCF7 cells, while Aurora A knockdown enhanced the sensitivity of MCF7AR cells (Fig. 2D,E). The data revealed that MCF7AR cells are responsive to the Aurora A inhibitor (Alisertib, Aurora Ai) with an IC50 of 2 μM, and this sensitivity decreases in RB-knockdown MCF7AR cells (Fig. 2F). These findings suggest that the cytotoxic impact of Aurora Ai is associated with the RB level in MCF7AR cells. Aurora A regulates the levels of p-SMC3 and p-H3, facilitating mitosis by promoting chromatin condensation and separation of sister chromatids, respectively (Fig. 2G,H). The Crystal Violet assay results demonstrated a significant increase in CDK4/6i sensitivity upon Aurora A knockdown (Fig. 2I). The levels of p-SMC3 and p-H3 decreased following Aurora A inhibitor treatment (Fig. 2J), accompanied by simultaneous reductions in fluorescence intensities of p-Aurora A and p-H3 with Aurora A inhibitor treatment (Fig. 2K).

### 3.3 Aurora A Inhibition Restores Sensitivity to CDK4/6i

**Figure 3:**
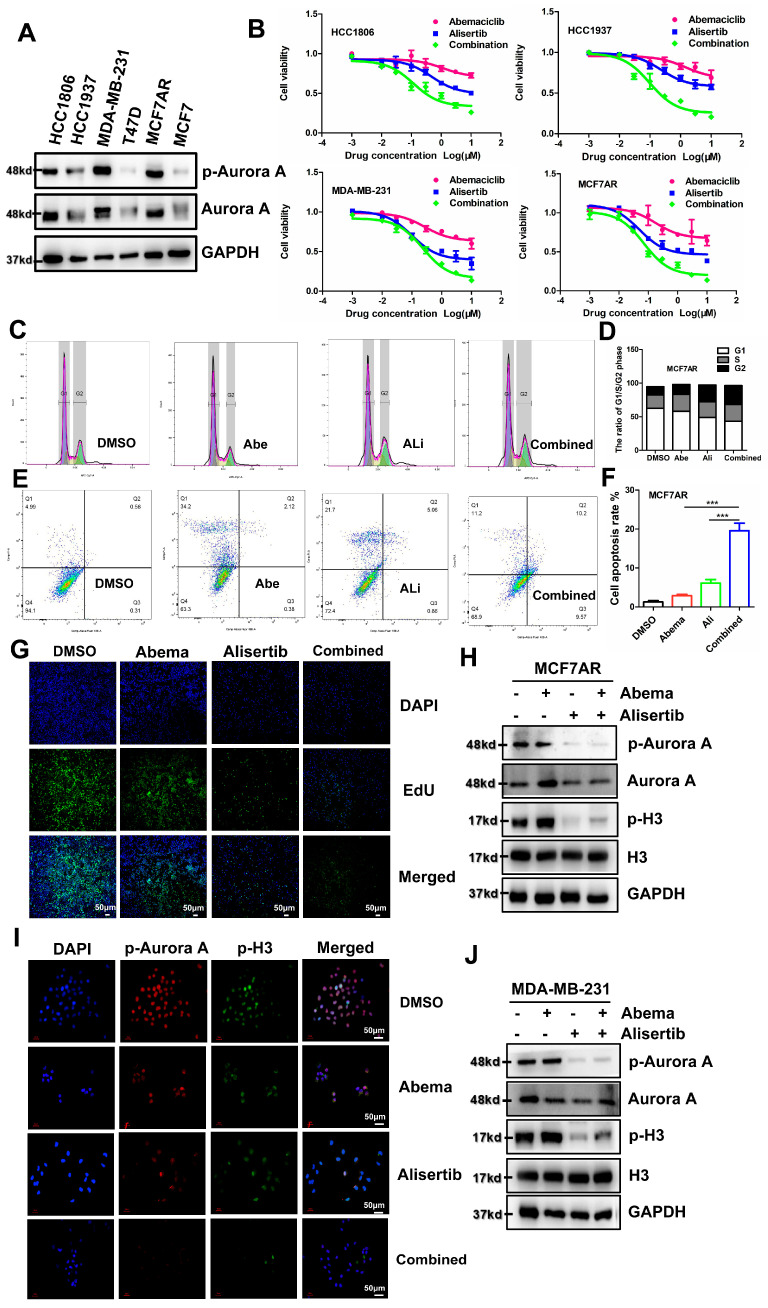
**Aurora A inhibition restores sensitivity to CDK4/6i.** (**A**) Protein levels of p-Aurora A/Aurora A and p-RB/RB were checked in six BC cell lines. (**B**) The synergistic effect of combined treatment with Abemaciclib and Alisertib was evaluated in HCC1806, HCC1937, MDA-MB-231, and MCF7AR cells. (**C**) The distribution of cells in G1, S, and G2 phases was checked by cell cycle assay following combined treatment. (**D**) The statistical chart illustrates the proportions of the G1, S, and G2 phases in each group. (**E**) Cell apoptosis was examined by flow cytometry. (**F**) The chart illustrates the proportions of cell apoptosis in each group. (**G**) The EdU assay was used for the detection of DNA replication stagnation, the fluorescence intensity of EdU indicated cell cycle progression. (**H**) Changes in levels of p-Aurora A/Aurora A and p-H3/H3 were assessed by WB when combined treatment in MCF7AR cells. (**I**) The fluorescence intensities of p-Aurora A and p-H3 were measured following treatment with Abemaciclib and Alisertib. (**J**) Protein level changes in p-Aurora A and p-H3 were examined following combined treatment in MDA-MB-231 cells. The results presented have been repeated in 3 biological replicates. Data, means ± SEM, ***p < 0.001.

We found that Aurora A was upregulated in MCF7AR cells compared to parental MCF7 cells, and its activation was associated with CDK4/6i resistance. High levels of p-Aurora A and p-RB were observed in certain breast cancer cell lines, and those with elevated p-Aurora A were more sensitive to alisertib. The combination of abemaciclib and alisertib demonstrated a notable cytotoxic effect (Fig. 3A,B). Cell cycle analysis revealed an increase in G2-phase cells post-combined treatment, indicating G2-phase arrest (Fig. 3C,D). Flow cytometry results showed a significant rise in cell apoptosis (Fig. 3E,F). EdU assay results indicated a halt in DNA replication following the combined treatment, likely due to cell cycle arrest (Fig. 3G). Phosphorylated histone H3 serves as a crucial marker for cell proliferation and is closely associated with mitotic processes. Western blot data displayed alterations in p-Aurora A and p-H3 levels following the combined treatment in MCF7AR cells (Fig. 3H). The fluorescence intensities of p-Aurora A and p-H3 concurrently decreased with the combined treatment (Fig. 3I). Protein level changes in p-Aurora A and p-H3 following combined treatment in MDA-MB-231 cells (Fig. 3J).

### 3.4 USP22 Mediates Stabilization of Aurora A

**Figure 4:**
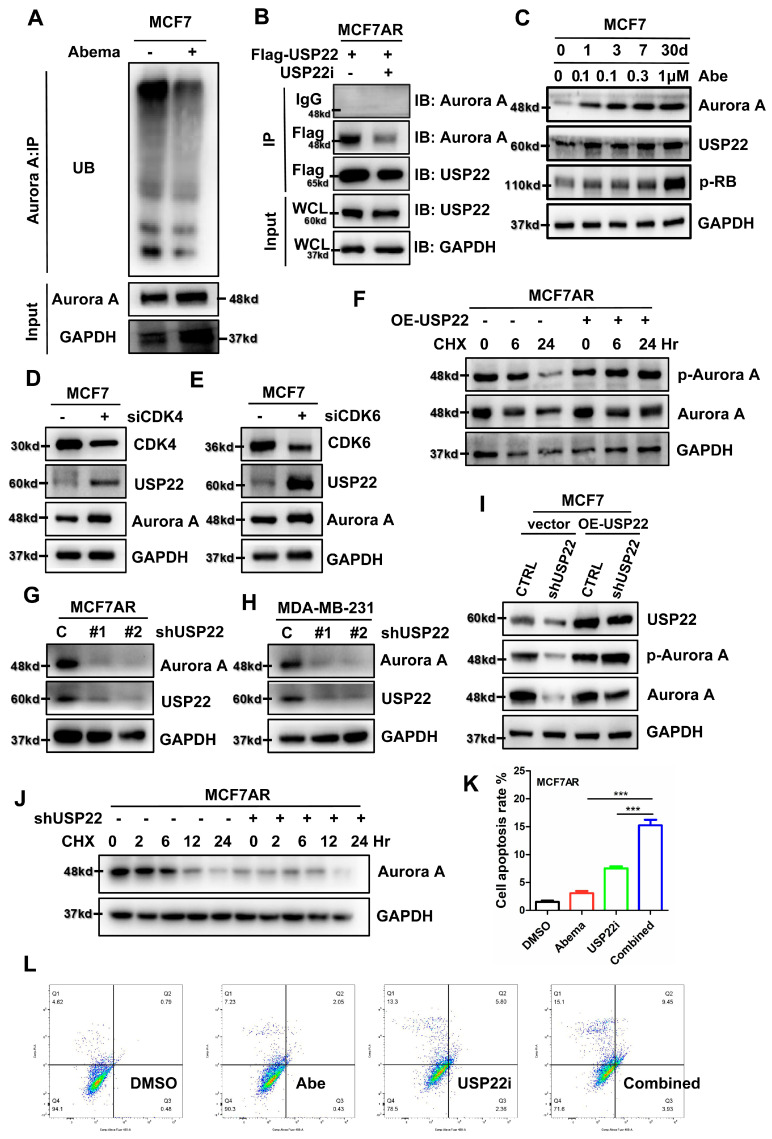
**USP22 mediates stabilization of Aurora A.** (**A**) Ubiquitination of Aurora A was detected following immunoprecipitation (IP) using an Aurora A antibody. (**B**) A Flag-USP22 plasmid was constructed, and a Flag antibody was used for the IP assay; USP22 and Aurora A proteins were detected upon USP22 inhibition. (**C**) MCF7 cells treated with Abemaciclib at five different time points during the AR screening process were analyzed for protein levels of Aurora A and p-RB. (**D**) Protein levels of Aurora A and USP22 were assessed following knockdown of CDK4. (**E**) Protein levels of Aurora A and USP22 were assessed following knockdown of CDK6. (**F**) Cycloheximide (CHX) was used to inhibit protein synthesis, and the degradation rate of Aurora A was measured at 0, 6, and 24 h post-CHX treatment. (**G**) Aurora A expression was evaluated after USP22 silencing in MCF7AR cells. (**H**) Aurora A expression was checked after USP22 silencing in MDA-MB-231 cells. (**I**) Aurora A protein levels were checked following USP22 overexpression in shUSP22-MCF7 cells. (**J**) Aurora A expression was monitored in shUSP22-MCF7AR cells treated with CHX in a time-dependent manner. (**K**,**L**) Cell apoptosis was confirmed by flow cytometry after treatment with CDK4/6i and USP22i. The results presented have been repeated in 3 biological replicates. Data, means ± SEM, ***p < 0.001.

To further investigate the mechanism by which Aurora A is highly expressed in CDK4/6i-resistant cells, we conducted an IP assay to detect the ubiquitination of Aurora A. The results suggested that CDK4/6i treatment reduced Aurora A ubiquitination in MCF7 cells (Fig. 4A). USP22, a deubiquitinating enzyme of interest in our laboratory, was found to interact with the Aurora A protein. Inhibition of USP22 blocked this protein interaction (Fig. 4B). To study the changes in Aurora A protein levels during the development of resistant strains, we subjected MCF7 cells to stepwise dose-increasing drug treatment (from 0.1 to 20 μM) over 6 months. We analyzed abemaciclib-treated MCF7 cells at five time points during the AR screening process and observed elevated levels of Aurora A and p-RB as drug resistance increased (Fig. 4C). Treatment with CDK4/6i led to increased Aurora A levels, and upregulation of USP22 was observed, along with a slight increase in Aurora A in CDK4 or CDK6 knockdown cells (Fig. 4D,E). The use of CHX to inhibit protein synthesis revealed that USP22 slowed the rate of Aurora A degradation (Fig. 4F). Silencing of USP22 reduced the expression of Aurora A (Fig. 4G,H). Overexpression of USP22 in shUSP22-MCF7 cells confirmed a correlation between the levels of Aurora A and USP22 (Fig. 4I). USP22 was found to regulate the expression of Aurora A in a time-dependent manner following CHX treatment (Fig. 4J). Flow cytometry results indicated an increase in apoptosis after treatment with CDK4/6i and USP22i (Fig. 4K,L).

### 3.5 Aurora A Inhibition Enhances the Anti-Tumor Effect of CDK4/6i In Vivo

**Figure 5:**
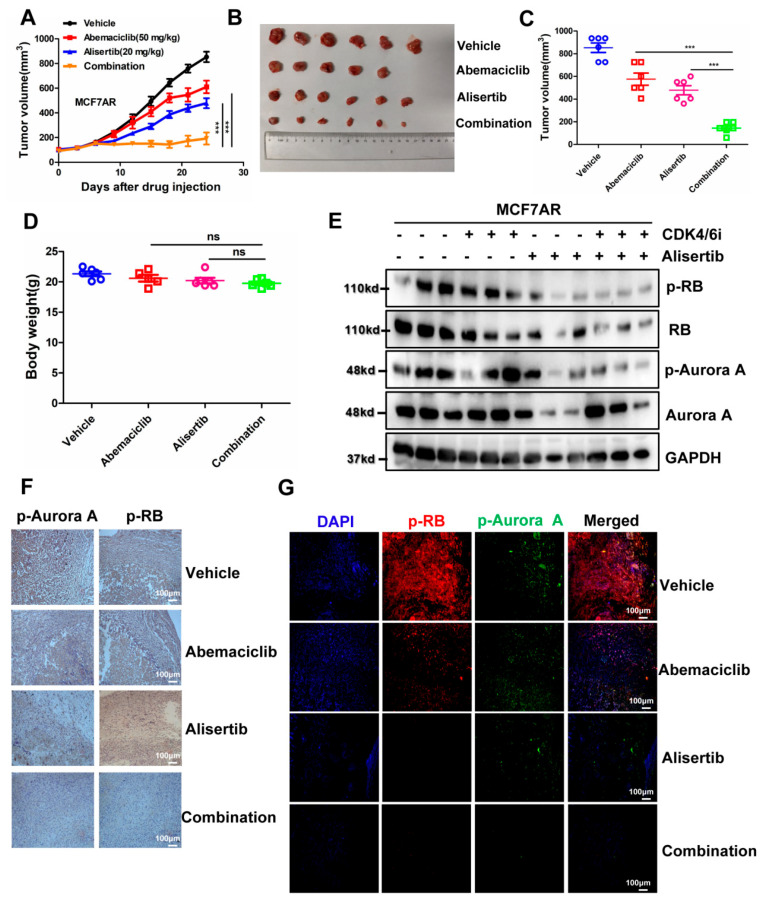
**Inhibiting Aurora A enhances the anti-tumor effect of CDK4/6i *in vivo*.** (**A**–**C**) MCF7AR cells were used to establish a nude mouse model. The mice were randomly divided into vehicle-, Abemaciclib-, Alisertib-, and combined-group after tumor size reached 100 mm^3^. Abemaciclib (50 mg/kg) or Alisertib (20 mg/kg) was administered through intragastric injection. Tumor volume was measured with a ruler and recorded. The tumor volume results were plotted on a chart, and representative tumor images were shown. (**D**) The tumor weights of the mice in each group were measured to assess the safety of the drugs. (**E**) Three samples from each group’s tumor tissue were analyzed by Western blot to detect the expression of p-RB/RB and p-Aurora A/AuroraA. (**F**) An immunohistochemical (IHC) assay was performed to assess the p-RB and p-Aurora A expression. (**G**) Multiplex immunofluorescence (mIF) assays were used to evaluate p-RB and p-Aurora A expression, with four representative images shown for each group. The results presented have been repeated in 3 biological replicates. Data, means ± SEM, ns, no significance; ***p < 0.001.

Aurora A kinase was proven to play a crucial role in CDK4/6i resistance, and Aurora A inhibition can enhance the sensitivity of CDK4/6i *in vitro*. The preclinical efficacy needs to be verified; MCF7AR cells were used for the nude mouse model. The mice were randomly divided into vehicle-, Abemaciclib-, Alisertib-, and combined-group when the tumor size reached 100 mm^3^. Each group had at least 6 nude mouse, the 50 mg/kg of Abemaciclib or 20 mg/kg of Alisertib was injected through intragastric administration in the nude mice. The data suggested that combined therapy of Abemaciclib and Alisertib showed a synergistic anti-tumor effect in the xenograft model, causing the tumor volume to shrink rapidly (Fig. 5A–C). The tumor weights of the mice in each group remained unchanged (Fig. 5D). The samples derived from the animal tumor tissue in 4 groups were used to detect the expression of p-RB/RB and p-Aurora A/AuroraA by Western Blot (Fig. 5E). Furthermore, we used IHC assay to detect the p-RB and p-Aurora A expression (Fig. 5F). The data from mIF assays indicated that p-RB and p-Aurora A were co-expressed and co-localized in four representative groups, The co-distribution of p-RB and p-Aurora A has obvious characteristics of regional consistency, the results showed that the expression level of p-RB and p-Aurora A decreased after the combined therapy (Fig. 5G).

### 3.6 High P-RB/P-Aurora A Expression Confers CDK4/6i Resistance in Breast Cancer

**Figure 6:**
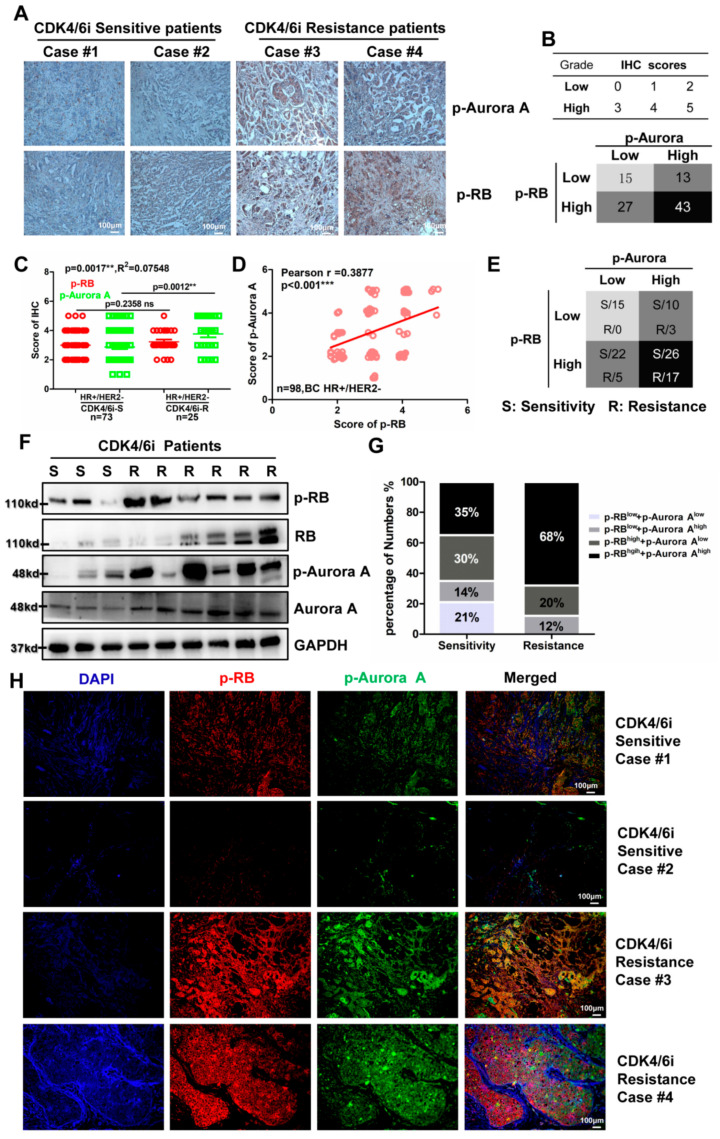
**High p-RB and p-Aurora A expression confers CDK4/6i resistance in breast cancer.** (**A**) The expression levels of p-RB and p-Aurora A were assessed by IHC assay in CDK4/6i-sensitive and resistant patients. (**B**) The expression profiles of p-RB and p-Aurora A in 98 patients are shown in the figure below. (**C**) The expression levels of p-RB and p-Aurora A in the CDK4/6i-sensitive and resistant groups were plotted in a scatter plot. (**D**) The correlation between p-Aurora and p-RB expression was presented as a scatter plot. (**E**) The number of CDK4/6i-sensitive and resistant patients was identified separately within each of the four categories. (**F**) The expression levels of p-RB and p-Aurora A in fresh tumor tissues from both CDK4/6-sensitive and resistant patients were checked by Western Blot. (**G**) Based on the expression levels of p-RB and p-Aurora A, the composition ratios of these four subgroups in CDK4/6i-sensitive and resistant patients are shown. (**H**) The expression of p-RB and p-Aurora A was examined by mIF assays, with representative images obtained from both CDK4/6i-sensitive and resistant patients. The results presented have been repeated in 3 biological replicates. Data, means ± SEM, ns, no significance; **p < 0.01; ***p < 0.001.

Although Abemaciclib has achieved good clinical benefits in the treatment of HR+/HER2- breast cancer, some patients experienced disease recurrence due to primary or secondary resistance [5]. We collected paraffin sections of breast cancer patients who have received CDK4/6i treatment from Renmin Hospital of Wuhan University. We found that p-RB and p-Aurora A were abnormally highly co-expressed in breast cancer cells, but the expression and co-localization of p-RB/p-Aurora A in breast cancer tissues are unknown. The IHC results showed that HR+/HER2- patients with high “p-RB/p-Aurora A” expression showed CDK4/6i resistance (Fig. 6A). The immunohistochemical scores were obtained through double-blind scoring by the pathologists, ranging from 0 to 5 points. They are classified as low expression with scores ranging from 0 to 2, and high expression ranging from 3 to 5. The expression profiles of the 98 patients with p-RB and p-Aurora A are shown in the figure below (Fig. 6B). The data suggusted that the expression of p-RB and p-Aurora A in CDK4/6i resistance group were higher than in sensitive group overallly (Fig. 6C), and the expression of p-Aurora A was positively correlated with p-RB in BC patients (Fig. 6D). Further, the number of sensitive and resistant patients was separately identified within each of the four categories (Fig. 6E). We examined the expressions of p-RB and p-Aurora A in fresh tumor tissues from both sensitive and resistant CDK4/6i BC patients, the data suggusted that they are highly-expressed in CDK4/6i BC patients (Fig. 6F). According to the expression of p-RB and p-Aurora A, the samples were divided into four groups: p-RB^L^+p-Aurora A^L^, p-RB^L^+p-Aurora A^H^, p-RB^H^+p-Aurora A^L^, and p-RB^H^+p-Aurora A^H^. The data showed that the p-RB^L^+p-Aurora A^L^ group was almost classified into the CDK4/6i-sensitive clinical patients. On the contrary, the CDK4/6i-resistant group includes the majority of p-RB^L^+p-Aurora A^H^ and p-RB^H^+p-Aurora A^H^ subgroups, which accounted for about 88% (Fig. 6G). The data from mIF assays indicated that p-RB and p-Aurora A were co-expressed and co-localized in four representative breast cancer patients, including four representative CDK4/6i-sensitive or resistant patients, respectively. The co-distribution of p-RB and p-Aurora A has obvious characteristics of regional consistency (Fig. 6H). Based on these results, we concluded that high p-RB/p-Aurora A expression may confer CDK4/6i resistance in HR+/HER2- BC patients.

### 3.7 The Mechanism Diagram of the Aurora A Activation Involved in CDK4/6i Resistance

To better understand the molecular mechanism described in this study, a schematic diagram was created to illustrate how highly active Aurora A kinase overcomes the SAC delay and promotes mitosis. High levels of phosphorylated RB were observed in CDK4/6 inhibitor-resistant cell lines, which induced SAC-mediated mitotic delay. Tumor cells with elevated Aurora A activity survived the drug screening process by overcoming the SAC delay and advancing the cell cycle. Inhibition of Aurora A in RB-phosphorylated, CDK4/6i-resistant cells may produce a synthetic lethal effect. Conversely, CDK4/6i-sensitive cells typically exhibit low levels of phosphorylated RB, and the low activity of Aurora A in these cells is insufficient to bypass cell cycle arrest.

## 4 Discussion

Although CDK4/6 inhibitors have shown significant clinical benefits in treating breast cancer, not all patients derive advantages from them, leading to primary and secondary drug resistance. Primary resistance occurs when patients with breast cancer show minimal response to the initial CDK4/6i treatment. While most patients initially respond well, disease progression eventually sets in, known as secondary drug resistance. Therefore, understanding the mechanism of CDK4/6i resistance is crucial to help clinicians optimize treatment strategies. Analyzing the characteristics of key molecules involved in CDK4/6i resistance is essential. These molecules can act as predictive biomarkers to identify potentially beneficial patient populations and new therapeutic targets to improve CDK4/6i efficacy.

**Figure 7:**
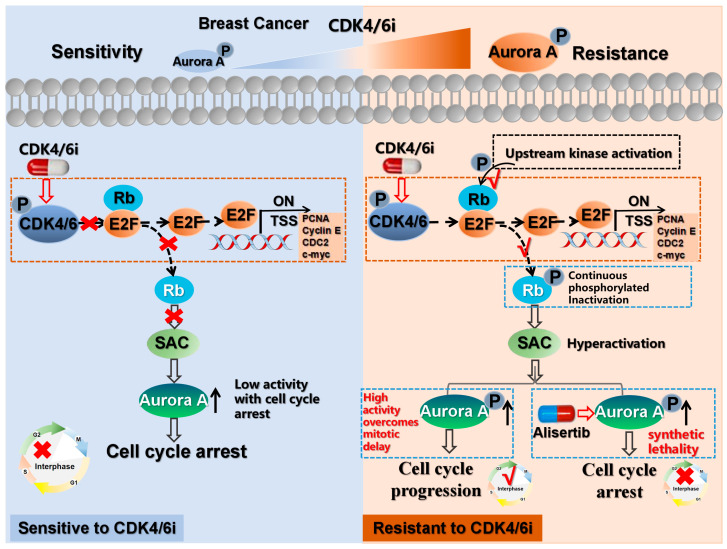
**The mechanism diagram of Aurora A activation involved in CDK4/6i resistance.** Elevated levels of phosphorylated RB and Aurora A were observed in CDK4/6i-resistant breast cancer cells. CDK4/6i treatment may block RB phosphorylation in CDK4/6i-sensitive cells, and continuous RB phosphorylation was found in resistant cells. The high levels of phosphorylated RB may activate the SAC effect, leading to mitotic delay and cell cycle arrest. Aurora A confers CDK4/6i resistance by overcoming SAC-induced delay and promoting mitosis. Inhibition of Aurora A in RB-inactivated, CDK4/6i-resistant cells may achieve a synthetic lethal effect.

Several potentially valuable targets and inhibitors have been discovered to address CDK4/6i resistance [19,37,38,39]. Several studies have also reported the role of PARP1 in mediating CDK4/6i resistance and driving the progression [40]. Activation of CDKs plays a significant role as an oncogenic factor in breast cancer [41]. We are striving to explore the key proteins on which breast cancer cells resistant to CDK4/6i rely for cell cycle regulation. To further understand how molecules evolve during the CDK4/6i resistance process, we obtained MCF7AR-resistant cell lines by gradually increasing the drug concentration in the CDK4/6i-sensitive cell line MCF7. The process of constructing the resistant cell lines lasted approximately six months, with the abemaciclib concentration ranging from 0.1 to the final 20 μM. We discovered that CDK4/6i-resistant cell lines with high phosphorylated RB expression may inactivate RB and SAC, leading to mitotic delay. The phosphorylation status of RB in drug-resistant cells is not affected by CDK4/6i and remains continuously inactivated. What kind of force drives drug-resistant cell strains to bypass the SAC checkpoint? Highly phosphorylated Aurora A has been found in CDK4/6i-resistant cell lines and patient samples, and its high activity facilitates the passage of drug-resistant cells through cell cycle arrest. Targeting Aurora A in combination with abemaciclib can exert synergistic antitumor effects in both cellular and animal models. The reason why Aurora A becomes abnormally activated during the development of drug resistance was further investigated. We found that increased Aurora A expression was inhibited by CDK4/6 during the development of resistance, and the upregulated Aurora A protein was regulated by USP22, the activity of which was inhibited by CDK4/6. The continuous upregulation of phosphorylated RB triggers the SAC effect and the development of the USP22-Aurora A pathway when CDK4/6i treatment is used, leading to increased Aurora A activity and overcoming cell cycle arrest. The mechanism diagram of the Aurora A activation involved in CDK4/6i resistance was illustrated in Fig. 7. It showed that highly active Aurora A kinase can overcome the SAC delay and promote mitosis. Therefore, the expression levels of Aurora A in the cell cycle must be investigated.

Taken together, our results indicate that inhibiting Aurora A in RB-inactivated CDK4/6i-resistant cells can induce a synthetic lethal effect. Clinical data suggest that patients with HR+/HER2- breast cancer with high expression of p-RB/p-Aurora A may exhibit resistance to CDK4/6i. This study demonstrated that RB inactivation may enhance the SAC checkpoint and cause mitotic delay, although it did not result in cancer cell apoptosis. Some researchers have observed a similar phenomenon where RB function is activated post-CDK4/6i treatment, leading to cancer cells entering a “senescent” state akin to dormancy without undergoing apoptosis [42]. Cancer cells release senescence signaling molecules that activate the EGFR survival pathway, enabling them to evade apoptosis. This suggests that tumor cells can adaptively respond to the RB state to evade apoptosis. Tryptophan metabolism has been linked to aging, and recent reports have implicated its involvement in TNBC progression [43]. Loss of NF1 function increases tumor cell sensitivity to CDK4/6 inhibitors. Moreover, targeting the vulnerability of cancer cells to hyperactivation of CDK4/6 kinase enhances the synthetic lethal effect [44]. NF1 loss also acts as a biomarker for predicting the efficacy of CDK4/6i targeted therapy. Tumor cells re-enter the cell cycle post-CDK4/6i treatment, limiting the long-term efficacy of the drug. The absence of TP53 can enhance p130 phosphorylation by CDK2, enabling tumor cells to overcome cell cycle arrest and promote tumor progression. Given that the P53 status can influence the sensitivity of colorectal cancer to ferroptosis, it is possible that inactivation of P53 is worthy of attention in CDK4/6i resistance [45,46]. Integrating these data, there seems to be a consensus that the absence or inactivation of tumor suppressor genes drives resistance to CDK4/6i through their respective regulatory mechanisms in the cell cycle.

The activity of Aurora A significantly increased during the formation of a CDK4/6i-resistant steady state, in which tumor cells rely on the high activity of Aurora A to overcome the mitotic delay effect caused by SAC activation. This results in the addition of Aurora A kinase activity to the tumor cells. Conversely, Aurora A inhibition leads to cancer cell fragility. Alisertib monotherapy has been reported to show good clinical activity and manageable safety in patients with BC with endocrine or CDK4/6i resistance [47]. Therefore, it may be necessary to check the RB status of each patient with CDK4/6i resistance. In patients with inactivated or absent RB, tumor cells may be more dependent on Aurora A activity, and Aurora Ai treatment may improve outcomes. Studies have indicated that the RB status may be a biomarker for CDK4/6i resistance in breast cancer and poor prognosis [48].

This study has several limitations. First, significant heterogeneity exists within tumors. Resistance mechanisms may involve multiple factors, which could undermine the effectiveness of targeting Aurora A alone to achieve the desired outcome. Second, the preclinical models used have inherent limitations. In this study, the cell resistance model was developed by gradually increasing the drug concentration; however, in clinical settings, medication dosages are typically administered more consistently. Additionally, organoid or patient-derived xenograft (PDX) models may better replicate the tumor microenvironment. Furthermore, the number of clinical samples analyzed was insufficient; a larger sample size is necessary to robustly support the current conclusions. Finally, the combined treatment protocol was evaluated through animal experiments to verify its efficacy. However, there are no established standards for optimizing treatment dosage and timing, and potential toxic side effects have not been thoroughly investigated. Therefore, a detailed analysis of drug resistance mechanisms is required, along with comprehensive data on drug dosage and safety in preclinical models, to support the clinical translational potential of targeting Aurora A to overcome resistance to CDK4/6i.

## 5 Conclusion

In this study, we demonstrated that Aurora A activation mediates resistance to CDK4/6i. Highly active Aurora A kinase facilitates the progression of drug-resistant cells through cell cycle arrest by overcoming SAC delay. In RB-inactivated, CDK4/6i-resistant cells, Aurora A inhibition may induce a synthetic lethal effect. Clinical data suggest that patients with high Aurora A expression may exhibit resistance to CDK4/6i. This study presents preclinical data on Aurora A inhibitors in breast cancer, highlighting their potential to expand therapeutic options by reversing CDK4/6i resistance.

## Data Availability

The mentioned database website in this paper was listed as http://gepia.cancer-pku.cn/.

## Supplementary Materials

The supplementary material is available online at https://www.techscience.com/doi/10.32604/or.2026.081653/s1.
